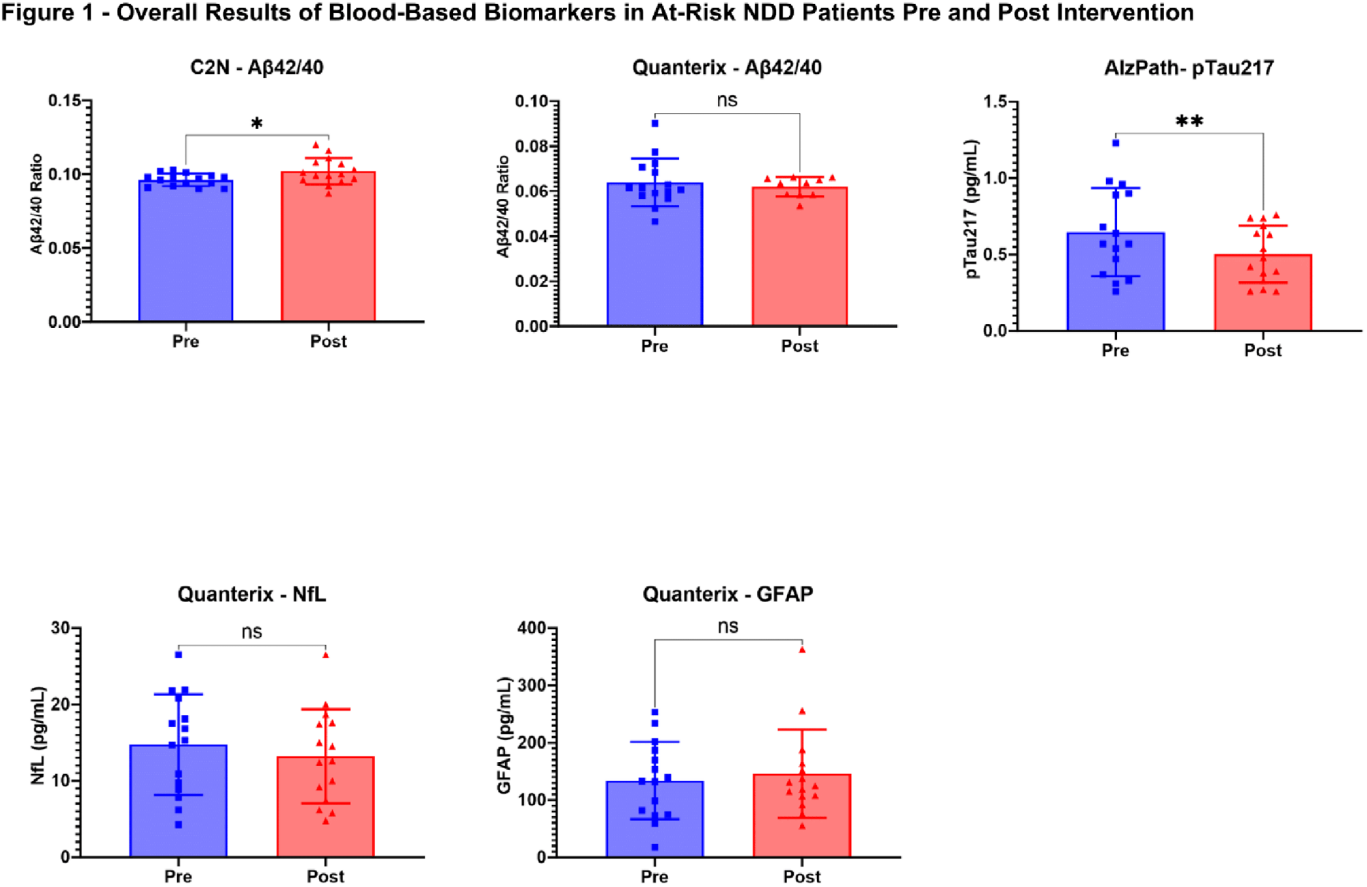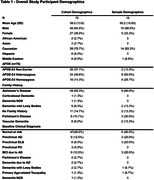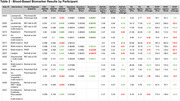# Blood‐based Biomarkers to Evaluate the Effectiveness of Individualized Alzheimer’s Risk Reduction Care: The Future of Preventive Neurology?

**DOI:** 10.1002/alz.088292

**Published:** 2025-01-09

**Authors:** Kellyann Niotis, Philip Sisser, Shannon Helfman, Christopher Janney, Hollie Hristov, Alon Seifan, Juan Melendez, Praveen Parthasarathy, Michael McCullough, Maia Mosse, Diana Saville, Helena Colvee, Corey Saperia, Danny Angerbauer, Jessica P. Lakis, Hanna Huber, Guglielmo Di Molfetta, Nicholas J. Ashton, Henrik Zetterberg, Richard S. Isaacson

**Affiliations:** ^1^ The Institute for Neurodegenerative Diseases (IND) Florida, Boca Raton, FL USA; ^2^ FAU Charles E. Schmidt College of Medicine, Boca Raton, FL USA; ^3^ Jersey Memory Assessment Service, St Helier, UK Jersey; ^4^ Renaissance School of Medicine at Stony Brook University, Stony Brook, NY USA; ^5^ University of California, San Francisco School of Medicine, San Francisco, CA USA; ^6^ Stanford University School of Medicine, Stanford, CA USA; ^7^ BrainMind, Cambridge, MA USA; ^8^ University of Miami School of Nursing and Health Studies, Miami, FL USA; ^9^ The Atria Institute, New York, NY USA; ^10^ Institute of Neuroscience and Physiology, University of Gothenburg, Mölndal Sweden; ^11^ Institute for Neurodegenerative Diseases (IND) Florida, Boca Raton, FL USA

## Abstract

**Background:**

Alzheimer's disease (AD), Dementia with Lewy Bodies (DLB), and other neurodegenerative diseases (NDD) develop over an extended preclinical period, sharing common risk factors and underlying pathophysiological mechanisms. Plasma proteins, including Amyloid‐beta peptides (Aβ) and Tau isoforms, facilitate differential diagnosis of NDD in their earliest stages, allowing for timely delivery of targeted interventions. Blood‐based biomarkers may also serve as a reliable means of monitoring disease progression and evaluating the effectiveness of individualized interventions across the spectrum of disease.

**Method:**

Participants (≥25 years) with a family history of AD/NDD with no or minimal neurological complaints, and those with a prior diagnosis of AD/NDD, are recruited for the Biomarker Repository for Alzheimer’s & Neurodegenerative Diseases (BioRAND) Study. Demographics, expanded medical history, APOE genotype, cognitive assessments, and NDD biomarkers including Aβ42/40 ratio, pTau217, Neurofilament light (NfL), and glial fibrillary acidic protein (GFAP) (via venipuncture and/or at‐home finger prick card testing) are collected. Clinical and/or research biomarkers include the commercially‐available C_2_N liquid‐chromatography‐tandem mass spectrometry amyloid assay, AlzPath SIMOA pTau217 assay, and Quanterix SIMOA amyloid, NfL and GFAP assays, which are collected at baseline and longitudinally. Participants have the option of sharing clinical data pertaining to NDD risk reduction interventions prescribed by their physicians.

**Result:**

Recruitment is ongoing (n=75) with 42 participants (56%) having biomarkers from ≥2 timepoints. Demographics and participant characteristics are summarized in Table 1. Fifteen (20%) participants provided clinical data with biomarkers measured before and after risk reduction interventions. Interventions included multi‐modal lifestyle recommendations (e.g., exercise, nutrition), FDA‐approved anti‐amyloid immunotherapies, pharmacological therapies to address modifiable risk factors (e.g., tirzepatide, rosuvastatin, escitalopram, hormone replacement therapy, suvorexant), and proposed geroprotective agents (e.g., rapamycin). Significant improvements (paired t‐test) were found on C_2_N Aβ42/40 ratio (p<0.05) and AlzPath pTau217 (p<0.005). Biomarker results in Table 2

**Conclusion:**

Evaluating the effectiveness of individualized AD/NDD risk reduction care is feasible with blood‐based biomarkers. Improvements in amyloid and tau were observed across the preclinical to clinical spectrum of NDD. Further study in a large diverse cohort is warranted to determine the clinical utility of blood‐based biomarkers in preventive neurology care.